# Possible delayed effect of unemployment on suicidal rates: the case of Hungary

**DOI:** 10.1186/1744-859X-13-12

**Published:** 2014-04-23

**Authors:** Konstantinos N Fountoulakis, Xenia Gonda, Peter Dome, Pavlos N Theodorakis, Zoltan Rihmer

**Affiliations:** 1 3rd Department of Psychiatry, School of Medicine, Aristotle University of Thessaloniki, 6, Odysseos Str. (1st Parodos Ampelonon Str.), Pylaia, Thessaloniki 55535, Greece; 2 Department of Clinical and Theoretical Mental Health, Faculty of Medicine, Semmelweis University, Budapest 1089, Hungary; 3 Laboratory for Suicide Research and Prevention, National Institute of Psychiatry and Addictions, Budapest H-1135, Hungary; 4 Department of Pharmacodynamics, Semmelweis University, Budapest H-1089, Hungary; 5 MTA-SE Neuropsychopharmacology and Neurochemistry Research Group, Hungarian Academy of Sciences, Semmelweis University, Budapest 1089, Hungary; 6 Mental Health Hospitals Trust of Attica "Dafni & Dromokaiteio", Athens 12462, Greece; 7 WHO National Counterpart for Mental Health, Athens 12462, Greece

**Keywords:** Suicide, Austerity, Delayed effect

## Abstract

**Background:**

During the last few years, many countries in Europe suffered from a severe economic crisis which resulted in high unemployment rates. In this frame, the possible relationship between unemployment rate and suicidal rates at the level of the general population has been debated recently.

**Material and methods:**

The official data concerning completed suicides and unemployment rates from the Hungarian Central Statistical Office for the years 2000–2011 were used. The percentage of changes from the previous year in the unemployment rate and the suicidal rates concerning both the general and the unemployed populations was calculated. Pearson correlation coefficient between the change in suicidal rates and change in unemployment rates was calculated both for the same year as well as after 1–6 years.

**Results:**

The correlations between the unemployment rate and suicide rates were strongly negative both for the general and for the unemployed populations (-0.65 and -0.55, respectively). The correlation of unemployment change with suicidality change after 1–6 years gave a peak strong positive correlation at 5 years for the general population (0.78). At 4 years after the index year, there is a peak correlation with a moderate value for the unemployed population (0.47) and a similar moderate value for the general population (0.46).

**Discussion:**

The current findings from Hungary suggest that unemployment might be associated with suicidality in the general population only after 3–5 years. It is possible that the distressing environment of the economic crisis increases suicidality in the general population rather than specifically in unemployed people.

## Background

Completed suicide rates vary from 3.5/100,000 in Greece to 24/100,000 in Hungary and more than 60/100,000 in certain east European countries (e.g. Belarus) [1]. During the last few years, many countries in Europe suffered from a severe economic crisis which resulted in high unemployment rates. In spite of the fact that clinical studies consistently show that unemployment is a risk factor for suicide [2,3], the possible relationship between unemployment rate and the suicidal rate at the level of the general population has been debated recently with conflicting opinions and conclusions [4-8].

Besides a positive association between suicide and unemployment reported from several European countries and from the USA [9,10], the overall conclusion was rather negative concerning the short-term and the presence of a robust relationship, especially concerning Greece and Italy [5,6]. The experience from other countries which underwent similar problematic periods is also inconclusive (e.g. Argentina and the Eastern Europe). At the same time, we should bear in mind that these studies are ecological (with obvious shortcomings) and also that the interpretations of the raw data are somewhat conflicting [6,9,11-13].

The so-called ‘negative cycle of poverty and mental ill health’ is well established in high-income countries. People with mental health problems are more likely to be pushed into poverty through increased health costs, loss of employment, reduced work hours and stigma [14]. However, the opposite (i.e. unemployment leads to psychiatric disorders and eventually suicide) has not been strongly proven. Yet, some results—mainly derived studies from populations who lost their job in mass lay-offs—suggest such a causation [15]. Furthermore, results of a meta-analysis of studies with individual-based data suggest that becoming unemployed is associated with greater levels of distress, while the opposite process (to move into employment) is associated with decreasing levels of distress [16]. In addition, some results from studies that used individual-level data also suggest that the current crisis leads to an increasing prevalence of mental disorders (i.e. major depression) which are also the most important risk factors for suicidal behaviour [17]. Furthermore, results from some of these studies also suggest that the detrimental effects of the crisis are more pronounced in those individuals who are involved personally (e.g. those who experienced job loss apropos of the crisis) [17]. In this frame, it is interesting to monitor any changes in suicidal rates in those countries under severe socio-economic stress.

During the last 25 years, in most European countries, the suicidal rates were either dropping or stable [1]. It is important to see if the adverse economic situation and especially unemployment decelerate or ever reverse this trend.

Recently, we have demonstrated that the very explicit 20-year-lasting decline of the Hungarian suicide rates had a turning point in 2006, while the unemployment rate increased in two steps (a smaller in 2005 and a bigger in 2009). In the same period, the annual antidepressant consumption (a possible proxy for the treatment of depression which is the most powerful risk factor for suicide) has doubled between 2000 and 2011 [18]. The current article tries to clarify the possible relationship between unemployment and suicide rate in Hungary through the last decade and during the ongoing economic crisis which is characterized by recession and high unemployment rates.

## Material and methods

The authors gathered the official data concerning completed suicides and unemployment rates from the Hungarian Central Statistical Office ( http://www.ksh.hu/) for the years 2000–2011. The percentage of changes from previous year in unemployment rate and the suicidal rates concerning both the general and the unemployed populations was calculated. Pearson correlation coefficient between the change in suicidal rates and the change in unemployment was calculated both for the same year as well as between unemployment and suicidality after 1–6 years. This means that the values of change in unemployment were matched with changes in suicidality after 1–6 years, and thus, six different correlation coefficients were calculated.

## Results

**Figure 1:**
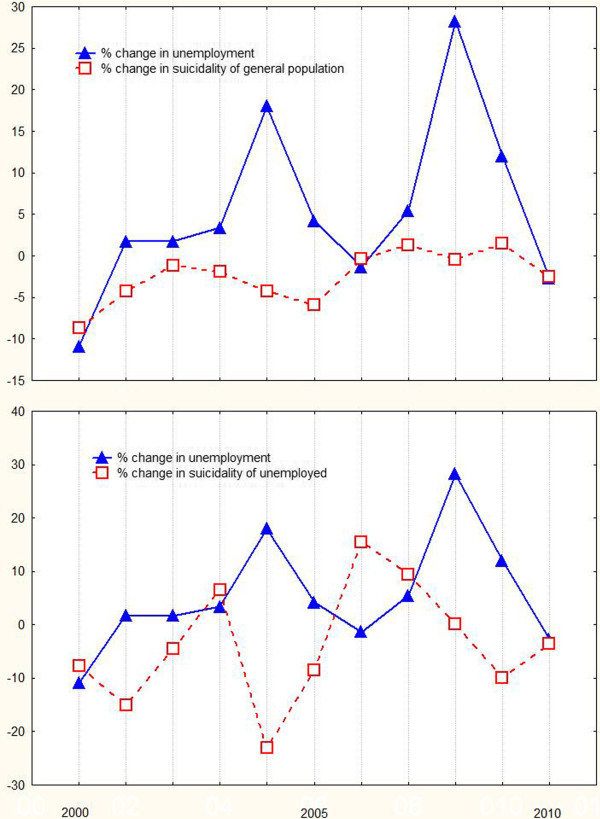
**Charts of change suicidality across the years in both populations.** General population (upper chart) and unemployed population (lower chart in comparison with unemployment change in both charts.

**Figure 2:**
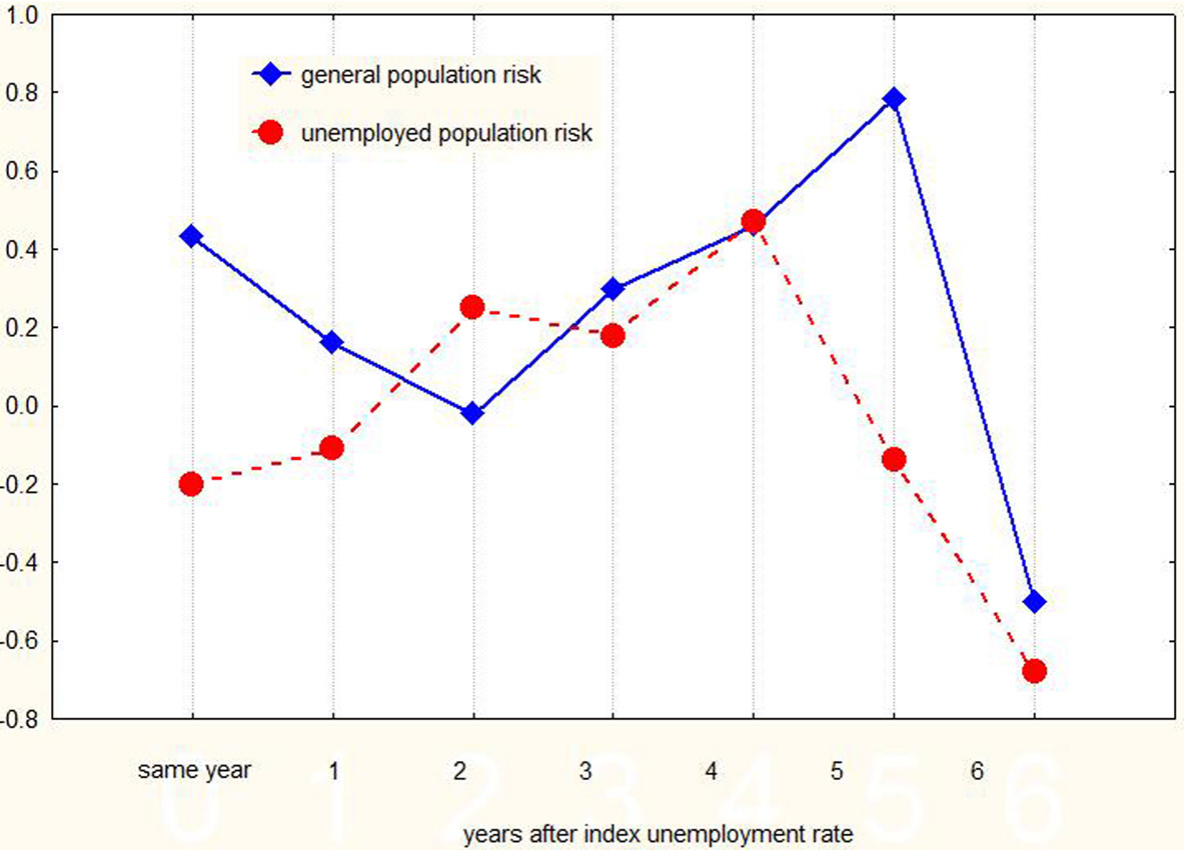
**Line plot of correlations between unemployment change and suicidality change in both populations vs. time.** It is evident that the correlation peaks several years after the index unemployment value.

The suicide rates were dropping steadily both in the general and the unemployed populations until 2006 when they reached a nadir, and since then, with some oscillations, are rather stable with a tendency to increase (Table 1). The correlation between the general population suicidal rates and the unemployment population suicidal rates was strong (0.91), but the correlation between the percent changes in suicidality in these populations was moderate (0.49). The correlations between unemployment rates and suicide rates were strongly negative both for the general and for the unemployed populations (-0.65 and -0.55, respectively). In Table 2, we present the matrix which shows the matching of change in unemployment rates with change in suicides in the general population during a period of 1–6 years. A similar matrix was created for the unemployed population also. The correlation of unemployment change with the suicide change after 1–6 years gave a peak strong positive correlation at 5 years for the general population (0.78). At 4 years after the index year, there is a peak correlation with a moderate value for the unemployed population (0.47) and a similar moderate value for the general population (0.46). These values were followed with negative correlations for the next years (Table 3). The charts of change in unemployment and suicide rates across the years in the general and the unemployed population are shown in Figure 1. The correlations between unemployment rate change and the suicide rate change in the two populations several years after the index unemployment value are shown in Figure 2.

**Table 1:** Unemployment and suicidal rates in both populations and percent changes from the previous year

**Year**	**Population**	**Suicide rate**		**Unemployment rate**	**Percent change in unemployment from previous year**	**Percent change in suicidality from the previous year**	
		**General population**	**Unemployed population**			**General population**	**Unemployed population**
2000	10,043,224	32.02	117.9	6.4			
2001	10,200,298	29.23	108.9	5.7	-10.94	-8.71	-7.63
2002	10,174,853	27.98	92.5	5.8	1.75	-4.28	-15.06
2003	10,142,362	27.65	88.3	5.9	1.72	-1.18	-4.54
2004	10,116,742	27.12	94.1	6.1	3.39	-1.92	6.57
2005	10,097,549	25.98	72.4	7.2	18.03	-4.20	-23.06
2006	10,076,581	24.44	66.3	7.5	4.17	-5.93	-8.43
2007	10,066,158	24.35	76.6	7.4	-1.33	-0.37	15.54
2008	10,045,401	24.67	83.8	7.8	5.41	1.31	9.40
2009	10,030,975	24.56	83.9	10	28.21	-0.45	0.12
2010	10,014,324	24.92	75.6	11.2	12.00	1.47	-9.89
2011	9,985,722	24.29	72.9	10.9	-2.68	-2.53	-3.57

**Table 2:** Matrix of matching of percent change in unemployment with percent change in suicidality

**Year**	**Percent change in unemployment from the previous year**	**Percent change in suicidality from the previous year (general population)**						
		**Same year**	**After 1 year**	**After 2 years**	**After 3 years**	**After 4 years**	**After 5 years**	**After 6 years**
2000								
2001	-10.94	-8.71	-4.28	-1.18	-1.92	-4.20	-5.93	-0.37
2002	1.75	-4.28	-1.18	-1.92	-4.20	-5.93	-0.37	1.31
2003	1.72	-1.18	-1.92	-4.20	-5.93	-0.37	1.31	-0.45
2004	3.39	-1.92	-4.20	-5.93	-0.37	1.31	-0.45	1.47
2005	18.03	-4.20	-5.93	-0.37	1.31	-0.45	1.47	-2.53
2006	4.17	-5.93	-0.37	1.31	-0.45	1.47	-2.53	
2007	-1.33	-0.37	1.31	-0.45	1.47	-2.53		
2008	5.41	1.31	-0.45	1.47	-2.53			
2009	28.21	-0.45	1.47	-2.53				
2010	12.00	1.47	-2.53					
2011	-2.68	-2.53						

This data is from the general population during a period of 6 years. A similar matrix was created concerning the unemployed population.

**Table 3:** Pearson correlation coefficients between index unemployment change and suicidality change after 1–6 years (latency)

**Latency years**	**General population**	**Unemployed population**
0	0.43	-0.20
1	0.16	-0.11
2	-0.02	0.25
3	0.30	0.18
4	0.46	0.47
5	0.78	-0.14
6	-0.50	-0.68

The coefficients concerning the general population are based on the figures shown in Table 2 and for the unemployed population on a similar table.

## Discussion

The current findings from Hungary suggest that unemployment might be associated with suicide rate in the general population only after 3–5 years. In the unemployed population, itself, the association is much attenuated and is restricted to the fourth year. The interpretation of these results suggests that prolonged unemployment may primarily lead to increased suicidality, and surprisingly, much of it might be unrelated to the experience of unemployment itself. Rather the distressing environment of the economic crisis increases suicidality (or decelerates the reduction rate) in the general population rather than specifically in unemployed people.

This possible explanation should be the focus of research because it implies that no single and reasonable factor like unemployment could be considered as having a causal relationship with suicidality [3]. A recent meta-analytic review showed that long-term unemployment is associated with increased suicide mortality, and the risk is greatest in the first 5 years [19].

Although it is believed that both attempts and completed suicides increase in periods of crisis [7], the experience from other countries that underwent problematic periods is inconclusive. Argentina experienced a severe crisis during 1999–2002, and according to the World Health Organization data, it experienced a decrease in suicidal rates the decade before the crisis (6.4–6.6 for the years 1985–1995) and an increase during 2000–2008 (7.5–7.7), but still, this increase was lower than the rates reported for the years before 1980 (9.4–9.9). Concerning the east European countries, the data are conflicting. For example, Hungary experienced an increase in suicide rates from 1955 until the mid-1980s, some years before the fall of the Berlin wall, and a marked decrease between 1986 and 2011 [18], while Ukraine experienced a sharp increase only after the Berlin wall had fallen.

It is evident that there is an urgent need for intensive screening, follow-up and treatment of people with suicidal ideation. The actions to be taken are also of prime importance. Since it is reported that the majority of suicide victims die by their first attempt [2,20], targeting suicide prevention beyond the framework of healthcare could be the only realistic option for suicidal prevention. A recent systematic review by the first author of the current article showed that only community networking is effective in reducing the actual number of suicides, while training of gatekeepers and other ‘educational-type’ campaigns have little or no effect at all [21].

A substantial decline of suicide rates throughout Europe, the USA and Canada happened in the last 20–25 years [22]; however, with the global economic crisis, the future of these rates is unknown. The current paper suggests that there is a time lag in the increase of suicidality, and vulnerable populations (probably mentally ill persons) remain to be more precisely identified.

Targeted interventions of proven efficacy should be applied, including specific psychoeducational programs. They should utilize long-term and repeated intervention, as well as community networking, and should not be restricted to gatekeepers' training and general (theoretical) education of the public.

The major flaw of the current paper is that (1) the degree of overlapping of unemployed populations across different years is unknown, so the measured data on changes of unemployment rates in index years and changes of unemployment suicide rates in the given years after index year(s) may come from only partially overlapping unemployed populations. (2) Because of the small number of observations (years where the data come from), the testing of statistical significance of the presented correlations is meaningless. (3) In Hungary, the suicide mortality of the general population seems to have stabilized after 2006, and only small fluctuations are evident. However, this is not the case with the unemployed population. (4) In the current study, the general population and the unemployed population are overlapping, and part time unemployment is not included. (5) A cohort effect could be present and affected the results.

### Limitations

The paper includes the analysis of population data. This means that overlapping of groups is significant and impossible to pair for important variables at the raw-data level. The time series is limited to a decade. Longer time series are important.

### Implications

The results suggest that austerity might exert a latent effect on the suicide rate, while the effect of prolonged unemployment cannot be excluded. Short-term unemployment does not seem to have any effect on suicide rates.

## Competing interests

The authors declare that they have no competing interests.

## Authors’ contributions

KNF had the idea, did the analysis, wrote the first draft and finalized the manuscript. PD, XG, PT and ZR collected the data, interpreted the results, corrected the drafts and finalized the manuscript. All authors read and approved the final manuscript.
